# The renin-angiotensin receptor blocker azilsartan medoxomil compared with the angiotensin-converting enzyme inhibitor ramipril in clinical trials versus routine practice: insights from the prospective EARLY registry

**DOI:** 10.1186/s13063-015-1100-8

**Published:** 2015-12-19

**Authors:** Peter Bramlage, Roland E. Schmieder, Anselm K. Gitt, Peter Baumgart, Felix Mahfoud, Hartmut Buhck, Taoufik Ouarrak, Martina Ehmen, Sebastian A. Potthoff, for the EARLY Registry Group

**Affiliations:** Institut für Pharmakologie und präventive Medizin, Menzelstrasse 21, 15831 Mahlow, Germany; Universitätsklinikum Erlangen, Medizinische Klinik 4, Schwerpunkt Nephrologie/Hypertensiologie, Erlangen, Germany; Institut für Herzinfarktforschung GmbH, Ludwigshafen, Germany; Herzzentrum Ludwigshafen, Medizinische Klinik B, Ludwigshafen, Germany; Clemens-Hospital Münster, Klinik für Innere Medizin I, Münster, Germany; Universitätsklinikum des Saarlandes, Klinik für Innere Medizin III, Homburg/Saar, Germany; MedCommTools, Medical-Scientific Consultancy, Hannover, Germany; Takeda Pharma, Berlin, Deutschland; Universitätsklinikum Düsseldorf, Klinik für Nephrologie, Düsseldorf, Germany

**Keywords:** Randomized controlled trial (RCT), Azilsartan-medoxomil, Registry, Hypertension, Clinical practice

## Abstract

**Background:**

Patient characteristics and blood pressure-related outcomes in randomized clinical trials (RCTs) differ from clinical practice because of stringent selection criteria. The present study aimed to explore the relationship between clinical trials and clinical practice. We analyzed data from patients enrolled in the “Treatment with Azilsartan Compared to ACE-Inhibitors in Anti-Hypertensive Therapy” (EARLY) registry comparing blood pressure (BP) effects of the angiotensin receptor blocker (ARB) azilsartan medoxomil (AZL-M) with the angiotensin-converting enzyme (ACE) inhibitor ramipril between patients who met the eligibility criteria of a previous RCT and those who did not.

**Methods:**

Patients with primary arterial hypertension were consecutively enrolled from primary care offices in Germany into the EARLY registry in a 7:3 ratio for treatment with AZL-M or an ACE inhibitor, provided that they met the following criteria at baseline: 1) no antihypertensive treatment prior to inclusion or a non-renin-angiotensin system (RAS) based monotherapy; 2) initiation of treatment with either AZL-M or an ACE inhibitor alone. Analyses were performed to evaluate BP effects for patients in the EARLY registry who met the selection criteria of a prior RCT (RCT+) versus those who did not (RCT-).

**Results:**

Out of 3,698 patients considered, 1,644 complied with the RCT criteria (RCT+) while 2,054 did not (RCT-). RCT- patients (55.5 %) displayed a higher risk profile in terms of age and comorbidities, and a wider spectrum of BP values at baseline, as highlighted by the grades of hypertension and mean BP values. The proportion of patients who achieved target blood pressure control in the RCT+ group was significantly higher for AZL-M versus ramipril (64.1 versus 56.1 %; *P* < 0.01), in accordance with the result of the clinical trial. In the RCT- AZL-M group, the proportion of patients who met BP targets was lower (58.1 %) than in the RCT+ AZL-M group (64.1 %), whereas the proportion of patients with target BP values in the RCT- ramipril and the RCT+ ramipril groups was similar (57.7 versus 56.1 %). Thus, in contrast to results for the RCT+ group, in the RCT- group, the target BP attainment rate for AZL-M was not significantly superior to that for ramipril. However, the tolerability profile of AZL-M and ramipril was comparable in both populations. At the 12-month follow-up, death and stroke rates were low (≤0.5 %) and adverse events did not differ between the AZL-M and ramipril groups, irrespective of RCT eligibility.

**Conclusions:**

These data confirm that the EARLY population comprised a broader spectrum of hypertensive patients than RCTs, and the differences in patient characteristics were accompanied by disparate rates of blood pressure goal attainment. Overall, the validity of the RCT was demonstrated and confirmed in clinical practice with a broader range of patients with various comorbidities.

**Electronic supplementary material:**

The online version of this article (doi:10.1186/s13063-015-1100-8) contains supplementary material, which is available to authorized users.

## Background

There are a number of antihypertensive drugs such as beta-blockers, angiotensin-converting enzyme (ACE) inhibitors, angiotensin receptor blockers (ARBs), calcium channel blockers, and diuretics being used to treat high blood pressure [1]. While all drug classes are generally considered to be effective and safe with differing side effect profiles based on data from randomized controlled trials (RCTs), many patients who would not have been eligible for RCTs have to be treated in clinical practice. This happens because of the stringent inclusion and exclusion criteria that generally apply for enrollment in an RCT. For this reason, patients such as those with compromised renal function, after myocardial infarction, those who are particularly young or old (and many more) are underrepresented in RCTs, but have to be treated in clinical practice [2].

Azilsartan medoxomil (AZL-M) is a more recently approved ARB that has been intensively studied in a number of clinical trials [3–8]. A particular noteworthy trial compared the antihypertensive efficacy and safety of AZL-M to ramipril in patients with grade 1 to 2 hypertension [8]. The primary efficacy endpoint was the change in clinic trough, seated systolic blood pressure (BP) from baseline with ambulatory BP additionally provided. AZL-M 40 and 80 mg reduced both clinic systolic BP and mean ambulatory systolic BP significantly more than ramipril at a dose of 10 mg (clinic SBP −20.6 ± 0.9 with 40 mg and −21.2 ± 0.9 with 80 mg AZL-M versus −12.2 ± 0.9 with ramipril; *P* < 0.001 for both doses). Adverse events leading to discontinuation of treatment were less frequent with both doses of AZL-M (2.4 % and 3.1 %) compared with ramipril (4.8 %).

The “Treatment with Azilsartan Compared to ACE-Inhibitors in Anti-Hypertensive Therapy” (EARLY) registry was conducted in Germany and included patients who initiated treatment with either AZL-M or an ACE inhibitor at baseline [9]. In this analysis, patient characteristics, blood pressure control and safety in the EARLY registry were compared for patients who fulfilled the eligibility criteria for the recent RCT versus patients who did not.

## Methods

The EARLY registry was a prospective, observational, national, multicenter registry with a 12-month follow-up. Details of the study protocol have been published previously [9]. Patients with arterial hypertension who initiated single-agent treatment with either AZL-M or an ACE inhibitor in Germany, based on the treating physicians’ decision, were included in a ratio of seven (AZL-M) to three (ACE inhibitors). The ratio was chosen because there is an abundance of data on the safety and efficacy of ramipril from both RCTs and observational data. AZL-M, on the other hand, was newly introduced to the market at the time of this registry. Thus, we decided to imbalance recruitment in favor of AZL-M. Patient demographics were recorded at baseline and at 6- and 12-month follow-up visits. The protocol was approved by the independent international ethics committee in Freiburg and the ethics committee of the State Medical Council of Rheinland-Pfalz, Germany. Written informed consent was obtained from all patients.

### Selection of sites and patients

The registry was conducted in primary care offices in Germany. To represent the ambulatory treatment of hypertension, centers were selected from a database maintained at the Institut für Herzinfarktforschung, Ludwigshafen. Adult patients (≥18 years old) with essential arterial hypertension were consecutively enrolled, providing that they met the following two criteria: 1) no anti-hypertensive treatment prior to inclusion or a non-RAS based antihypertensive monotherapy; 2) monotherapy using AZL-M or any ACE inhibitor was initiated at baseline. Exclusion criteria were as follows: 1) treatment with antihypertensive drugs for an indication other than hypertension (for example, beta-blockers or diuretics for heart failure); 2) history of alcohol, drug abuse, or illegal drug addiction because of an expected lack of compliance with the registry requirements; 3) life expectancy of less than one year, 4) pregnancy or breast feeding; or 5) participation in other trials or registries. Patients with contraindications to any of the components of AZL-M or the ACE inhibitors selected were not permitted to enroll.

### Selection criteria

For the present analysis, patients included in the EARLY registry were divided into two groups according to the selection criteria used in an RCT by Bönner et al. (Table 1) that is the only trial available comparing AZL-M with an ACE inhibitor (ramipril) and thus close to the design chosen in EARLY [8]. Data were compared for patients who met the RCT selection criteria relative to those who did not. Further, patients receiving an ACE inhibitor other than ramipril were excluded.

**Table 1:** Selection criteria for the Bönner trial [8] and the corresponding criteria applied to the selection of EARLY patients

	Bönner et al. [8]	EARLY RCT+ population	Proportion of eligible patients (%)
Inclusion criteria			
Age	≥18 years	≥18 years	100.0
SBP	150–180 mmHg	150–180 mmHg	70.0
Laboratory profile	Not considered clinically significant	Not recorded	
Exclusion criteria			
SBP	>180 mmHg	>180 mmHg	7.9
DBP	>114 mmHg	>114 mmHg	2.6
Secondary hypertension	Excluded	As to SPC	
Severe renal disease; eGFR ≤30 ml/1.73 m^2^	Excluded	Renal insufficiency	2.8
Major CV event or intervention <6 month	Excluded	Stroke excluded	2.7
Sign. cardiac conduction defects	Excluded		
Aortic valve stenosis	Excluded	Not recorded	
Concomitant antihypertensive treatment or medication known to affect BP	Excluded	Excluded at baseline	32.5
Previous history of cancer not in remission for at least 5 years	Excluded	Malignancy excluded	2.6
Type 1 or poorly controlled type 2 diabetes mellitus (hemoglobin A1c >8.0 %)	Excluded	Hemoglobin A1c >8.0 % excluded; diabetes type not recorded	5.0
Hyperkalemia (serum potassium > upper limit of normal, 5.5 mmol/L)	Excluded	Potassium >5.5 mmol/L excluded	0.9
Night shift work	Excluded	Not recorded	
Pregnant or nursing women and woman of childbearing potential not using approved means of contraception	Excluded	Pregnant or nursing women were excluded	
Patients with alcohol or drug abuse	Not considered for exclusion	Excluded	
Treatment			
Pre-treatment	Not specified	Newly diagnosed or non-RAS monotherapy pretreatment	
Treatment	AZL-M vs. ramipril	AZL-M vs. ramipril; other ACEi excluded	

### Statistics

All summaries are based on the available data. Continuous variables were summarized with descriptive statistics (absolute numbers, means, standard deviations (SD), or medians with 25^th^ and 75^th^ percentiles), as appropriate. Categorical data were recorded as the number (n) and percentage (%) of patients in each category. Comparisons between groups were made with Pearson's chi-squared test for categorical variables and the Kruskal-Wallis test for continuous measures. Percentages were determined on the basis of the number of patients with data available for each respective parameter (that is, no percentages for missing values provided).

To evaluate BP differences between groups that differed at baseline, two multivariate models were used (Table 3). Model 1 adjusted for SBP/DBP at baseline. Model 2 adjusted for SBP/DBP at baseline, newly diagnosed or established hypertension, age, gender, and diabetes. Event rates (percentage) at one year are shown as raw and multivariate-adjusted data with the following variables considered: SBP/DBP at baseline, newly diagnosed or established hypertension, age, gender, and diabetes. Estimated mean differences were calculated with 95 % CI using a general linear model.

*P*-values ≤ 0.05 were considered to indicate statistically significant differences between groups. All *P*-values were calculated using two-sided tests. The statistical analysis was performed with SAS 9.2 (SAS Institute, Inc., Cary, NC, USA).

## Results

**Fig. 1:**
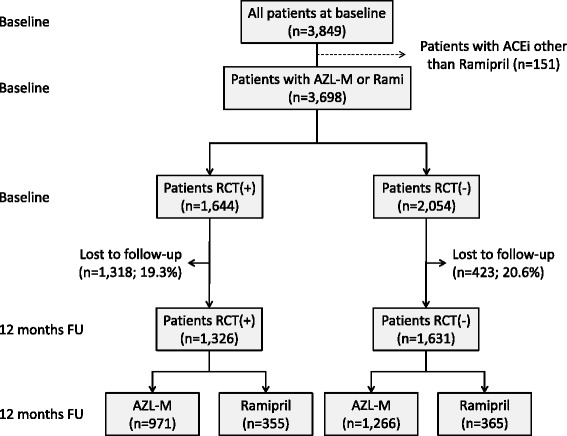
Patient inclusion and follow-up

The EARLY registry enrolled 3,849 patients who initiated treatment with either AZL-M or an ACE inhibitor. Only 151 patients received an ACE inhibitor other than ramipril, and they were subsequently excluded (Fig. 1). Of the remaining 3,698 patients, 1,644 complied with the inclusion and exclusion criteria of the RCT (RCT+) (Table 1) while 2,054 did not (RCT-). Follow-up was available for 1,326 patients in the RCT+ group (80.7 %) and 1,631 patients in the RCT- group (79.4 %). Patient characteristics for the RCT+ and RCT- groups with or without a 12-month follow-up are displayed in Additional file 1: Table S1, which illustrates any potential difference between those with or without a follow-up.

### Patient characteristics

At baseline, the mean age of 3,698 patients receiving either AZL-M or ramipril was 59.3 (±13.0) years, 46.6 % were female, and the mean body weight was 83.4 (±15.6) kg (Table 2). The mean SBP/DBP was 159.5 (±17.1)/93.5 (±10.5) mmHg, the majority of patients had grade 1 or 2 hypertension (75.6 %), and 6.0 % of patients had a BP of < 140/90 mmHg. Comorbidities included diabetes (19.4 %), coronary artery disease (9.6 %), chronic obstructive pulmonary disease (7.2 %), microalbuminuria (6.6 %), heart failure (5.7 %), renal disease (3.3 %), peripheral artery disease (2.9 %), and prior stroke/transient ischemic attack (2.7 %).

**Table 2:** Patient characteristics for the overall group and split by RCT eligibility at baseline with those receiving an ACE inhibitor other than ramipril excluded (n = 151)

	All patients (n = 3,698)	RCT(+) (n = 1,644)	RCT(−) (n = 2,054)	*P*-value
Age, years	59.3 ± 13.0	57.0 ± 12.8	61.2 ± 12.9	<0.0001
Female, %	46.6	44.0	48.7	<0.01
Body weight, kg	83.4 ± 15.6	83.9 ± 15.1	82.9 ± 16.0	<0.01
BMI, kg/m^2^	28.4 ± 4.7	28.3 ± 4.4	28.5 ± 5.0	<0.01
Hypertension (HT)				
Newly diagnosed HT	37.1	57.2	21.1	<0.0001
Office BP systolic, mmHg	159.5 ± 17.1	162.5 ± 9.5	157.0 ± 21.0	<0.0001
Office BP diastolic, mmHg	93.5 ± 10.5	94.8 ± 8.0	92.5 ± 12.1	<0.0001
Mean BP, mmHg	115.5 ± 10.9	117.4 ± 6.7	114.0 ± 13.2	<0.0001
Pulse pressure, mmHg	65.9 ± 15.2	67.7 ± 10.9	64.4 ± 17.8	<0.0001
BP <140/90 mmHg, %	6.0	0.0	10.8	<0.0001
Hypertension grade				
High normal	4.0	0.0	7.2	<0.0001
Grade 1	32.2	25.1	37.9	<0.0001
Any EOD*	62.8	53.9	66.9	<0.001
No EOD*	37.2	46.1	33.1	<0.001
Grade 2	43.4	60.0	29.8	<0.0001
Grade 3	18.5	14.8	21.5	<0.0001
Comorbidity				
Diabetes, %	19.4	15.3	22.6	<0.0001
Heart failure, %	5.7	3.0	7.9	<0.0001
CAD, %	9.6	5.2	13.2	<0.0001
Prior stroke/TIA, %	2.7	0.0	4.9	<0.0001
PAD, %	2.9	1.7	3.8	<0.001
COPD, %	7.2	6.4	7.9	0.09
Renal characteristics				
Known renal disease (%)	3.3	1.0	5.2	<0.0001
Microalbuminuria, %	6.6	3.4	9.1	<0.0001

*Legend: RCT* randomized controlled trial, *BP* blood pressure, *EOD* end-organ damage, *CAD* coronary artery disease, *TIA* transient ischemic attack, *PAD* peripheral artery disease, *COPD* chronic obstructive pulmonary disease. Values are indicated as percentage (%), median (interquartile range), or mean ± standard deviation; * any EOD is defined as any of diabetes, heart failure, CAD, stroke, PAD, known renal disease, microalbuminuria, or left ventricular hypertrophy

The RCT+ group was younger (*P* < 0.0001) and had a higher body weight (*P* < 0.01). Mean office SBP, DBP, mean BP, and pulse pressure were all higher in the RCT+ group (*P* < 0.0001 for all parameters), and none of the patients had a BP of < 140/90 mmHg compared with 10.8 % in the RCT- group (*P* < 0.0001). With respect to disease severity, the frequency of grade 2 hypertension was higher in the RCT+ group (60.0 % versus 29.8 %; *P* < 0.0001), whereas high-normal BP, and grade 1 and grade 3 hypertension were more frequent in the RCT- group (*P* < 0.0001 for all). Grade 1 hypertension with end-organ damage was less frequent in the RCT+ group (53.9 % versus 66.9 %; *P* < 0.001). The prevalence of chronic obstructive pulmonary disease was similar in the RCT+ (6.4 %) and RCT- groups (7.5 %; *P* = 0.09), but the prevalence of diabetes was lower in the RCT+ compared to the RCT- groups (15.3 % versus 22.6 %; *P* < 0.0001). All other comorbidities were less common in the RCT+ group as follows: coronary artery disease (5.2 % versus 13.2 %; *P* < 0.0001), prior stroke/transient ischemic attack (0.0 % versus 4.9 %; *P* < 0.0001), known renal disease (1.0 % versus 5.2 %; *P* < 0.0001), heart failure (3.0 % versus 7.9 %; *P* < 0.0001), microalbuminuria (3.4 % versus 9.1 %; *P* < 0.0001), and peripheral artery disease (1.7 % versus 3.8 %; *P* < 0.001; Table 2).

### BP control with AZL-M versus ramipril by RCT eligibility

For the RCT+ group, raw unadjusted data demonstrated that reductions in SBP, DBP, and mean pressure were 29.9 mmHg, 14.7 mmHg, and 19.8 mmHg, respectively, in the AZL-M group compared with 27.1 mmHg, 12.5 mmHg, and 17.4 mmHg in the ramipril group (*P* < 0.01, < 0.01, and < 0.001, respectively) (Table 3). The proportion of patients who attained a target BP of < 140/90 mmHg was 64.1 % in the AZL-M group compared with 56.1 % in the ramipril group (*P* < 0.01). Using data adjusted for baseline SBP/DBP (model 1), and baseline SBP/DBP, age, gender, and diabetes (model 2), AZL-M remained superior to ramipril for SBP, DBP, and mean BP (*P* < 0.01), and the percentage of patients with BP < 140/90 mmHg (*P* < 0.01).

**Table 3:** Blood pressure reductions: comparison of treatment groups (AZL-M versus ramipril) by RCT eligibility in those with 12-mo. follow-up

	RCT eligible				RCT not eligible			
	RCT(+) AZL-M value (95 % CI)	RCT(+) rami value (95 % CI)	Mean difference (95 % CI)	*P*-value	RCT(−) AZL-M value (95 % CI)	RCT(−) rami value (95 % CI)	Mean difference (95 % CI)	*P*-value
Raw (unadjusted)								
Δ Systolic, mmHg	29.9 (29.0–30.9)	27.1 (25.4–28.7)	2.8 (1.1–4.7)	<0.01	22.7 (21.5–23.9)	18.9 (16.9–21.0)	3.8 (1.3–6.3)	<0.01
Δ Diastolic, mmHg	14.7 (14.1–15.3)	12.5 (11.4–13.6)	2.2 (1.0–3.4)	<0.01	11.6 (10.9–12.3)	10.7 (9.4–12.0)	0.9 (−0.6–2.5)	0.07
Δ Mean pressure, mmHg	19.8 (19.2–20.4)	17.4 (16.3–18.5)	2.4 (1.2–3.6)	<0.001	15.3 (14.5–16.1)	13.4 (12.1–14.8)	1.9 (0.2–3.5)	<0.01
Δ Pulse pressure, mmHg	15.2 (14.4–16.1)	14.5 (13.1–16.0)	0.7 (−1.0–2.4)	0.37	11.1 (10.1–12.1)	8.3 (6.5–10.0)	2.8 (0.7–5.0)	<0.05
Δ Heart rate, bpm	3.9 (3.4–4.4)	3.7 (2.9–4.5)	0.7 (−0.7–2.0)	0.45	2.3 (1.6–2.9)	2.2 (1.1–3.4)	0.1 (−1.3–1.3)	0.79
BP <140/90 mmHg, %	64.1 (61.0–67.2)	56.1 (50.7–61.3)	n.a.	<0.01	58.7 (55.9–61.5)	57.7 (52.4–62.8)	n.a.	0.73
Model 1 (adjusted)								
Δ Systolic, mmHg	29.7 (28.9–30.4)	27.8 (26.5–29.0)	1.9 (0.4–3.4)	<0.05	22.0 (21.3–22.7)	21.6 (20.2–22.9)	0.4 (−1.1–1.9)	0.58
Δ Diastolic, mmHg	14.5 (14.1–15.0)	13.0 (12.2–13.8)	1.5 (0.6–2.5)	<0.01	11.4 (10.9–11.8)	11.6 (10.7–12.4)	−0.2 (−1.2–0.8)	0.68
Δ Mean pressure, mmHg	19.6 (19.0–20.1)	17.9 (17.0–18.7)	1.7 (0.7–2.6)	<0.001	15.0 (14.4–15.4)	15.0 (14.0–15.8)	0.0 (−1.0–1.0)	0.99
Δ Pulse pressure, mmHg	15.1 (14.5–15.8)	14.7 (13.7–15.9)	0.4 (−0.9–1.7)	0.57	10.6 (10.0–11.2)	10.0 (8.8–11.2)	0.6 (−0.7–2.0)	0.35
Δ Heart rate, bpm	3.9 (3.4–4.4)	3.7 (2.9–4.5)	0.2 (−0.7–1.1)	0.65	2.3 (1.9–2.8)	2.0 (1.2–2.8)	0.3 (−0.6–1.3)	0.48
BP <140/90 mmHg, %	64.1 (61.1–67.1)	56.0 (50.8–61.1)	n.a.	<0.01	58.7 (55.9–61.5)	56.6 (51.4–61.7)	n.a.	0.38
Model 2 (adjusted)								
Δ Systolic, mmHg	29.7 (28.9–30.5)	27.7 (26.4–28.9)	2.0 (0.6–3.5)	<0.01	22.0 (21.3–22.7)	21.4 (20.1–22.7)	0.6 (−0.9–2.1)	0.43
Δ Diastolic, mmHg	14.5 (14.0–15.0)	13.1 (12.3–13.9)	1.4 (0.5–2.4)	<0.01	11.1 (10.9–11.8)	11.6 (10.7–12.4)	−0.2 (−1.1–0.8)	0.67
Δ Mean pressure, mmHg	19.6 (19.1–20.1)	17.9 (17.1–18.8)	1.6 (0.7–2.6)	<0.01	14.9 (14.4–15.4)	14.8 (14.0–15.7)	0.1 (−0.9–1.1)	0.91
Δ Pulse pressure, mmHg	15.2 (14.6–15.9)	14.6 (13.5–15.7)	0.6 (−0.6–1.9)	0.35	10.7 (10.0–11.3)	9.8 (8.7–11.0)	0.8 (−0.5–2.1)	0.22
Δ Heart rate, bpm	3.9 (3.4–4.4)	3.7 (2.9–4.5)	0.2 (−0.7–1.1)	0.66	2.3 (1.9–2.8)	2.0 (1.2–2.8)	0.3 (−0.6–1.3)	0.47
BP <140/90 mmHg, %	64.4 (61.3–67.4)	55.7 (50.4–60.9)	n.a.	<0.01	59.4 (56.7–62.2)	56.3 (51.0–61.5)	n.a.	0.30

*Legend: AZL-M* azilsartan medoxomil, *ACEi* angiotensin-converting enzyme inhibitor, *rami* ramipril, *RCT* randomized controlled trial, *CI* confidence interval, *BP* blood pressure, *SBP* systolic blood pressure, *DBP* diastolic blood pressure, *n.a.* not applicable. To illustrate the adjusted changes in BP, three pretreatment BP values were chosen to represent the three borders between four quartiles; model 1: adjusted for SBP/DBP at baseline; model 2: adjusted for SBP/DBP at baseline (model 1), newly diagnosed or established hypertension, age, gender, and diabetes

For the RCT- group, raw unadjusted data indicated that reductions in SBP, DBP, mean pressure, and pulse pressure were 22.7 mmHg, 11.6 mmHg, 15.3 mmHg, and 11.1 mmHg, respectively, in the AZL-M group relative to 18.9 mmHg, 10.7 mmHg, 13.4 mmHg, and 8.3 mmHg, respectively, in the ramipril group (*P* < 0.01, 0.07, <0.01, and <0.05, respectively) (Table 3). The proportion of patients who attained target BP levels was similar in the AZL-M (58.7 %) and the ramipril groups (57.7 %; *P* = 0.73). Using models 1 and 2, no significant differences were observed between the two treatment groups for any parameter.

### Safety profile

After a 12-month follow-up (Table 4), the rate of death was 4 out of 971 (0.4 %) with AZL-M versus none out of 355 with ramipril in RCT+ patients (*P* = 0.23). There were no strokes in either group. Death rates were 0.5 % with AZL-M versus 0.3 % with ramipril in RCT- patients (*P* = 0.61).

**Table 4:** Safety during 1-year follow-up in patients receiving AZL-M or ramipril by RCT eligibility in those with 12-mo. follow-up

	RCT eligible				RCT not eligible			
Adverse events (AEs) at 1 year	RCT(+) AZL-M	RCT(+) rami	Mean difference (95 % CI)	*P*-value	RCT(−) AZL-M	RCT(−) rami	Mean difference (95 % CI)	*P*-value
Death, % (95 % CI)	0.4 (0.1–1.1)	0.0	n.a.	0.23	0.5 (0.2–1.0)	0.3 (0.0–1.5)	n.a.	0.61
Patients without AE, %	94.0	94.9	n.a.	0.53	92.0	91.0	n.a.	0.51
Patients with any AE, %	6.0	5.1	n.a.	0.53	8.0	9.0	n.a.	0.51
Laboratory values								
∆ HbA1c, %	0.02 ± 0.78	0.15 ± 0.72	−0.13 (−0.40–0.14)	0.35	0.01 ± 1.10	0.08 ± 1.06	−0.07 (−0.39–0.25)	0.69
∆ Glucose fasting, mg/dl	5.76 ± 77.68	1.90 ± 19.59	3.85 (−23.65–31.4)	0.91	0.12 ± 21.13	2.81 ± 15.17	−2.69 (−10.70–5.32)	0.37
∆ Creatinine, mg/dl	0.04 ± 0.17	0.01 ± 0.20	0.03 (−0.02–0.08)	0.09	−0.06 ± 1.06	−0.10 ± 1.14	0.04 (−0.22–0.31)	0.90
∆ Potassium, mmol/L	0.05 ± 0.39	0.04 ± 0.53	0.02 (−0.16–0.20)	0.69	0.00 ± 0.51	0.08 ± 0.54	−0.08 (−0.25–0.09)	0.42
∆ GFR, ml/min/1.73 m^2^	−2.26 ± 10.10	−0.05 ± 11.58	−2.21 (−5.26–0.84)	0.11	−1.45 ± 14.15	−1.08 ± 14.90	−0.37 (−3.86–3.12 )	0.69

*Legend: AZL-M* azilsartan medoxomil, *rami* ramipril, *AE* adverse event, *SAE* serious adverse event, *GFR* glomerular filtration rate, *n.a.* not applicable

The majority of patients in both patient groups, whether eligible for the RCT or not, had no adverse events reported during the 1-year follow-up (>90 %) (Table 4). This percentage was nominally lower in the RCT+ than in the RCT- group, but with no statistically significant differences between the AZL-M and ramipril subgroups. Furthermore, changes in laboratory values as outlined in Table 4 were clinically negligible, and no significant differences were observed for the AZL-M and ramipril groups, respectively.

## Discussion

RCTs act as the principal source of evidence-based data upon which recommendations in clinical guidelines are based. Therefore, an appreciation of the differences between patient demographics and treatment responses in RCTs compared with clinical practice is of great importance for the successful and widespread implementation of therapeutic strategies. In the present study, data from patients in the EARLY registry were grouped and analyzed according to their eligibility for enrollment in a previous RCT that compared AZL-M with ramipril [8]. The results 1) demonstrate that the EARLY patient population not meeting the RCT inclusion criteria (RCT-) displayed different baseline characteristics and blood pressure responses compared with those who did meet the inclusion criteria; 2) demonstrate that in patients not meeting the RCT inclusion criteria, the difference in the blood pressure lowering effect between AZL-M and ramipril was non-significant, at least after adjustment for baseline variables; and 3) confirmed the principal result of a strong blood pressure lowering efficacy of AZL-M in the order of 20 mmHg systolic and 11 mmHg diastolic.

### Patient selection

The use of selection criteria in RCTs results in a carefully selected patient population that is less heterogeneous than that seen by primary care physicians. Among patients in the EARLY registry, 55.5 % were not eligible for the Bönner RCT. Those patients not eligible for the RCT were older, had a lower body weight, and lower BP values. While the majority of patients in both the RCT+ and the RCT- group had grade 1 or 2 hypertension (85.1 % and 67.7 %, respectively), the RCT- group also contained patients with high-normal BP, and a greater proportion of patients with grade 1 hypertension with end-organ damage, as well as grade 3 hypertension. All comorbidities except for chronic obstructive pulmonary disease were more frequent in the RCT- group. In summary, the RCT- group displayed a higher risk profile in terms of age and comorbidities, and a wider spectrum of BP values at baseline, as highlighted by the grades of hypertension and the mean BP values. These findings are similar to those of other non-interventional studies; for example, the “Global Registry of Acute Coronary Events” (GRACE) demonstrated that RCT-eligible patients were a lower risk population compared with non-eligible patients [10]. In the prospective SERVE registry, which included previously treated patients with inadequately controlled hypertension in German clinical practice, 72 % of patients were not eligible for the corresponding RCT trial COACH [11].

### Blood pressure reduction

Data on blood pressure control in the present study indicate that for patients in the RCT+ group, AZL-M was superior to ramipril for SBP, DBP, and mean pressure, as well as the proportion of patients who met a target BP of < 140/90 mmHg. Adjustment for baseline SBP/DBP, and baseline SBP/DBP, newly diagnosed or established hypertension, age, gender, and diabetes did not influence these results. However, for patients in the RCT- group, while raw data suggested that AZL-M was likewise superior to ramipril for SBP, DBP, pulse pressure, and mean pressure, differences between the two treatment groups were not maintained after adjustment for confounding variables. Accordingly, the difference between AZL-M and ramipril in the proportion of patients who achieved a BP target of < 140/90 mmHg was not significant when using raw or adjusted data. Interestingly, the percentage of target BP attainment in the ramipril RCT+ and RCT- groups was consistent, ranging from 56 % to 58 % across all analyses (raw and adjusted data), whereas for AZL-M, the rate of target BP attainment was 64.1 % in the RCT+ group and 58.7 % in the RCT- group. This suggests that the selection criteria used in the previous RCT of AZL-M versus ramipril may have led to a slight overestimation of the blood pressure lowering capacity of AZL-M when compared to ineligible patients or patients in real-life clinical practice, respectively. The potential reasons for the lack of a difference between both groups may be caused by 1) the higher age and the longer duration of hypertension in the RCT- group; 2) the lower blood pressure at baseline with 11 % being already controlled; and 3) more patients with diabetes, coronary artery disease, prior stroke, and renal disease. On the other hand, the overall conclusion that AZL-M provides better blood pressure control than ramipril is not invalidated by the present analysis, because AZL-M remained numerically superior to ramipril for patients in the RCT- group. It is worth noting that the opposite effect was observed for the RCT COACH: the blood pressure lowering effect was underestimated compared with real-life data from the SERVE registry.

### Safety

Safety was assessed based on the recording of adverse events including death and stroke as well as by recording laboratory values. The overall percentage of any adverse events was very low with between 5.1 and 9.0 % reported for the duration of the 12-month observation. This is in substantial contrast to the RCT [8] where 38.1 % of patients in the AZL-M 40 mg group and 43.7 % of patients in the AZL-M 80 mg group reported any adverse event and 38.6 % of patients in the ramipril groups reported any adverse event. This occurred despite the fact that the RCT only had a follow-up of 22 weeks as opposed to 12 months in the current registry. This finding may reinforce the notion that observational registries, although designed to capture rare adverse events in real-world clinical practice, may fail to do so because of the negligence of physicians to actually report them.

### Limitations

Limitations of this analysis are related to the inherent differences between RCTs and registries reflecting clinical practice. In RCTs, monitoring and follow-up of patients tend to be more frequent, which may enhance compliance and persistence to treatment. In the Bönner et al. [8] RCT of AZL-M versus ramipril, patients were evaluated at 4-week intervals, whereas, in the EARLY registry, follow-up was conducted at 6 and 12 months. Another key difference is the lack of randomization in patient registries, with physicians being responsible for the decision to initiate therapy with one drug or another. This is accompanied by a lack of blinding, which would prevent a selection bias of patients based on their characteristics. Finally, though we adjusted for SBP/DBP at baseline, newly diagnosed or established hypertension, age, gender, and diabetes, we may have failed to adequately consider unknown bias which can only be alleviated by randomized controlled trials.

## Conclusions

The data confirm that the EARLY population comprised a broader spectrum of patients than the prior RCT [8], and the differences in patient characteristics were accompanied by differing percentages of blood pressure goal attainment. The data are important because they confirm the principal efficacy of AZL-M in clinical practice within a broader range of patients with various comorbidities.

## Additional file

Additional file 1: Table S1. Patient characteristics for the overall group with those receiving an ACE inhibitor other than ramipril being excluded (n = 151) and split by RCT eligibility at baseline and availability of the 12-month follow-up. (DOC 53 kb)

## Abbreviations

ACE: angiotensin-converting enzyme
ACEi: angiotensin-converting enzyme inhibitor
ARB: angiotensin receptor blocker
AZL-M: azilsartan medoxomil
BP: blood pressure
DBP: diastolic blood pressure
EARLY: Treatment with Azilsartan Compared to ACE- Inhibitors in Anti-Hypertensive Therapy
GRACE: Global Registry of Acute Coronary Events
RCT: randomized controlled trial
SBP: systolic blood pressure
SD: standard deviation
