# Supplementary material for: The renin-angiotensin receptor blocker azilsartan medoxomil compared with the angiotensin-converting enzyme inhibitor ramipril in clinical trials versus routine practice: insights from the prospective EARLY registry
Source: Trials. 2015 Dec 19;16:581. doi: 10.1186/s13063-015-1100-8 (PMC4684935; doi:10.1186/s13063-015-1100-8)
Supplement: Additional file 1: Table S1. — Patient characteristics for the overall group with those receiving an ACE inhibitor other than ramipril being excluded (n = 151) and split by RCT eligibility at baseline and availability of the 12-month follow-up. (DOC 53 kb) [file 13063_2015_1100_MOESM1_ESM.doc]

**Table S1:** Patients characteristics for the overall group with those receiving an ACE inhibitor other than Ramipril being excluded (n=151) and split by RCT eligibility at baseline and availability of the 12 months follow-up.

|  | RCT (+) with FU  (n=1326) | RCT (+) w/o FU  (n=318) | p-value | RCT (-) with FU  (n=1631) | RCT (-) w/o FU  (n=423) | p-value |
| --- | --- | --- | --- | --- | --- | --- |
| Age, years | 57.2 ± 12.6 | 55.9 ± 13.6 | 0.23 | 61.3 ± 12.9 | 60.9 ± 12.8 | 0.65 |
| Female, % | 44.7 | 40.9 | 0.22 | 50.7 | 40.9 | < 0.001 |
| Body weight, kg | 83.8 ± 15.1 | 84.5 ± 15.1 | 0.27 | 82.5 ± 15.9 | 84.8 ± 16.4 | < 0.01 |
| BMI, kg/m^2^ | 28.2 ± 4.4 | 28.4 ± 4.4 | 0.49 | 28.5 ± 5.1 | 28.6 ± 4.6 | 0.47 |
| Hypertension (HT) |  |  |  |  |  |  |
| Newly diagnosed HT | 58.7 | 50.9 | <0.05 | 20.8 | 22.0 | 0.61 |
| Office BP systolic, mmHg | 162.7 ± 9.4 | 161.9 ± 9.8 | 0.15 | 156.8 ± 20.7 | 157.7 ± 22.2 | 0.66 |
| Office BP diastolic, mmHg | 94.9 ± 7.9 | 94.4 ± 8.1 | 0.28 | 92.5 ± 12.0 | 92.6 ± 12.6 | 0.78 |
| Mean BP, mmHg | 117.5 ± 6.7 | 116.9 ± 7.0 | 0.10 | 113.9 ± 13.0 | 114.3 ± 14.1 | 0.62 |
| Pulse pressure, mmHg | 67.8 ± 11.0 | 67.4 ± 10.8 | 0.53 | 64.3 ± 17.7 | 65.0 ± 17.8 | 0.71 |
| BP <140/90 mmHg, % | 0.0 | 0.0 | -- | 10.5 | 12.2 | 0.31 |
| Hypertension grade |  |  |  |  |  |  |
| High normal | 0.0 | 0.0 | -- | 6.9 | 8.4 | 0.28 |
| Grade 1 | 24.4 | 28.3 | 0.15 | 39.2 | 32.1 | < 0.01 |
| Any EOD* | 54.3 | 52.1 | 0.78 | 67.6 | 63.2 | 0.45 |
| No EOD* | 45.7 | 47.9 | 0.78 | 32.4 | 36.8 | 0.45 |
| Grade 2 | 60.6 | 57.5 | 0.31 | 29.3 | 31.9 | 0.32 |
| Grade 3 | 15.0 | 14.2 | 0.70 | 21.0 | 23.7 | 0.23 |
| Comorbidity |  |  |  |  |  |  |
| Diabetes, % | 16.0 | 12.6 | 0.13 |  |  |  |
| Heart failure, % | 3.5 | 0.9 | <0.05 | 7.8 | 8.4 | 0.67 |
| CAD, % | 5.7 | 3.2 | 0.07 | 13.1 | 13.6 | 0.79 |
| Prior stroke / TIA, % | 0.0 | 0.0 | -- | 4.5 | 6.4 | 0.13 |
| PAD, % | 1.8 | 1.3 | 0.49 | 3.9 | 3.3 | 0.59 |
| COPD, % | 7.1 | 3.6 | <0.05 | 8.9 | 3.5 | < 0.001 |
| Renal characteristics |  |  |  |  |  |  |
| Known renal disease, % | 1.0 | 0.9 | 0.96 | 5.4 | 4.4 | 0.41 |
| Microalbuminuria, % | 3.6 | 2.3 | 0.46 | 9.2 | 8.5 | 0.78 |

*Legend:* RCT, randomized controlled trial; BP, blood pressure; EOD, end-organ damage; CAD, coronary artery disease; TIA, transient ischemic attack; PAD, peripheral artery disease; COPD, chronic obstructive pulmonary disease. Values are indicated in percent (%), median (interquartile range), or mean±standard deviation; any EOD is defined as any of diabetes, heart failure, CAD, stroke, PAD, known renal disease, microalbuminuria or left ventricular hypertrophy.
